# Molecular characterization of a DNA fragment harboring the replicon of pBMB165 from *Bacillus thuringiensis *subsp. *tenebrionis*

**DOI:** 10.1186/1471-2164-7-270

**Published:** 2006-10-23

**Authors:** Junyan Huang, Suxia Guo, Jacques Mahillon, Géraldine A Van der Auwera, Li Wang, Dongmei Han, Ziniu Yu, Ming Sun

**Affiliations:** 1 State Key Laboratory of Agricultural Microbiology, College of Life Science and Technology, Huazhong Agricultural University, Wuhan 430070, Hubei, People's Republic of China; 2 Laboratory of Food and Environmental Microbiology, Université catholique de Louvain, Croix du Sud 2/12, B-1348 Louvain-la-Neuve, Belgium

## Abstract

**Background:**

*Bacillus thuringiensis *belongs to the *Bacillus cereus sensu lato *group of Gram-positive and spore-forming bacteria. Most isolates of *B. thuringiensis *can bear many endogenous plasmids, and the number and size of these plasmids can vary widely among strains or subspecies. As far as we know, the replicon of the plasmid pBMB165 is the first instance of a plasmid replicon being isolated from subsp. *tenebrionis *and characterized.

**Results:**

A 20 kb DNA fragment containing a plasmid replicon was isolated from *B. thuringiensis *subsp. *tenebrionis *YBT-1765 and characterized. By Southern blot analysis, this replicon region was determined to be located on pBMB165, the largest detected plasmid (about 82 kb) of strain YBT-1765. Deletion analysis revealed that a replication initiation protein (Rep165), an origin of replication (*ori165*) and an iteron region were required for replication. In addition, two overlapping ORFs (*orf6 *and *orf10*) were found to be involved in stability control of plasmid. Sequence comparison showed that the replicon of pBMB165 was homologous to the pAMβ1 family replicons, indicating that the pBMB165 replicon belongs to this family. The presence of five transposable elements or remnants thereof in close proximity to and within the replicon control region led us to speculate that genetic exchange and recombination are potentially responsible for the divergence among the replicons of this plasmid family.

**Conclusion:**

The replication and stability features of the pBMB165 from *B. thuringiensis *subsp. *tenebrionis *YBT-1765 were identified. Of particular interest is the homology and divergence shared between the pBMB165 replicon and other pAMβ1 family replicons.

## Background

*B. thuringiensis*, belonging to the *Bacillus cereus sensu lato *group, is a gram-positive and spore-forming bacterium that produces parasporal crystals during sporulation. The proteinaceous parasporal crystals show toxicity against insect larvae. The toxin protein genes, usually insecticidal crystal protein (ICP) genes, are mainly located on large conjugative plasmids [1].

*B. thuringiensis *strains appear to contain plasmid DNA in proportion of 10–20% of total cell DNA [2]. Generally, plasmids of different *B. thuringiensis *strains vary in number and size, with the number between 1–12 and the size from 2 to > 250 kb [3,4]. In addition to harboring ICP genes, *B. thuringiensis *plasmids can also bear insertion sequences and transposons that are commonly associated with crystal protein genes [5], or biosynthesis genes of the heat-stable toxin thuringiensin [6].

These plasmids consist of two groups: (1) the small plasmids (< 15 kb), which perform rolling-circle replication (RCR) using a single-stranded (ssDNA) intermediate [7], and (2) the large plasmids that normally follow a theta-replicating mode [8]. Theta replicons are currently divided into six groups (Group A-F) [9]. Although there have been relatively few studies focusing on the characterization of Gram-positive theta replicons, as opposed to their Gram-negative counterparts, plasmids pertaining to the broad-host-range pAMβ1 family (group D) have been mostly studied from Gram-positive bacteria [10-13]. To date, only four plasmids from the *B. cereus *group have been analyzed and reported to belong to the pAMβ1 family. Of these, p43 (65 kb) comes from *B. thuringiensis *subsp. *kurstaki *HD263, and a 2,828 bp replication region of p43 has been cloned [14]. The broad-host-range conjugative plasmid pAW63 (71,777 bp) has been isolated from *B. thuringiensis *subsp. *kurstaki *HD73, and a 4.1 kb replicon of pAW63 has been characterized [8]. pBT9727 (77,112 bp) is the sole plasmid in the pathogenic strain *B. thuringiensis *subsp. *konkukian *97-27, and its replication protein and the predicted origin have been analyzed by sequence comparison [15]. pXO2 (96,231 bp) is the second virulence plasmid of *Bacillus anthracis*, and a 2,429 bp replication region has been identified [16].

In this study, a DNA fragment was shown to contain a plasmid replicon capable of supporting replication and maintaining stability in *B. thuringiensis*. The location of this replicon was identified as being on pBMB165, the largest detected plasmid (about 82 kb) from *B. thuringiensis *subsp. *tenebrionis *YBT-1765. Sequence analysis revealed that the replicon of pBMB165 shared significant homology to the replicons of pAMβ1 family plasmids, suggesting that pBMB165 also belongs to this plasmid family. Moreover, the characterization of the replicon region of pBMB165 provided additional information on the evidence of genetic exchange, molecular evolution and distribution of these closely related plasmids in their various isolates of origin.

## Results and discussion

### Isolation and localization of a plasmid replicon from YBT-1765

**Figure 1:**
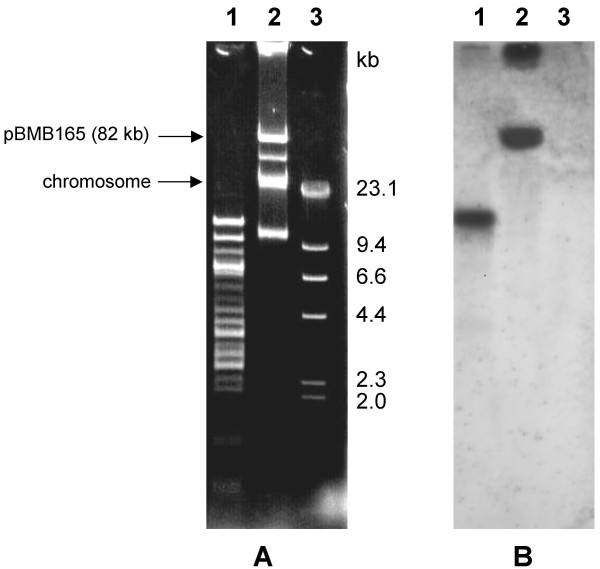
**Localization of a plasmid replicon in native plasmids from YBT-1765**. (A) Profile of agarose gel electrophoresis. (B) Southern blot with the 12 kb *Eco*RI fragment from pBMB1651 as probe. Lane 1, the total plasmid of YBT-1765 digested with *Eco*RI; Lane 2, the total plasmid of YBT-1765; Lane 3, λ *Hin*dIII marker.

The total plasmid DNA of YBT-1765 was digested by *Eco*RI. After gel electrophoresis, 2–12 kb fragments were separately excised from the gel and purified by DNA gel extraction. The purified plasmid fragments were then cloned separately into the *Eco*RI site of the replicon cloning vector pDG780. The resulting recombinant plasmids were electroporated into *B. thuringiensis *subsp. *kurstaki *plasmid-cured strain BMB171. Only one Km^R ^transformant was selected and contained a recombinant plasmid designated pBMB1651 (with a 12 kb insert), and then the recombinant plasmid pBMB1651 was introduced into the CaCl_2_-treated *E. coli *competent cell for further sequence analysis. On the basis of the above result, it was therefore believed that a plasmid replicon was located within the cloned 12 kb *Eco*RI fragment. To identify the plasmid harboring the 12 kb *Eco*RI replication fragment, a Southern blot experiment was performed, in which the 12 kb *Eco*RI fragment of pBMB1651 was used as probe. The result showed that this replication fragment was located on the largest plasmid detected in YBT-1765, named pBMB165 (about 82 kb, Fig. 1).

### Identification and characterization of the minireplicon

**Figure 2:**
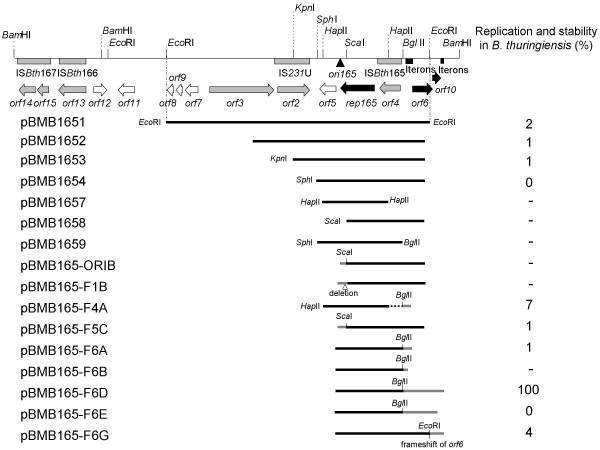
**Physical map of the 20 kb fragment cloned from pBMB165 and determination of the minimal replicon**. The predicted ORFs are shown by block arrows indicating the direction of transcription. The putative transposable elements are shown by hatched blocks. Replication-related ORFs, an origin of replication (*ori165*) and iterons region are in black. Other ORFs are shown by white block arrows. The stabilities of the different derivatives in *B. thuringiensis *are indicated. Inability of the constructs to replicate in *B.thuringiensis *is indicated by '-'. The fragments obtained by PCR amplification are indicated by the gray lines. The deletion in Rep165 is shown by the open triangle. The frame-shift introduced in *orf6 *is shown.

To further pinpoint the location of the pBMB165 minireplicon, a series of deletion derivatives was constructed and tested for replication in *B. thuringiensis *(Fig. 2). Six of the resulting plasmids, pBMB1652 (an 8.0 kb *Sau3*AI fragment from the 12 kb *Eco*RI fragment of pBMB1651 inserted into the *BamH*I site of pDG780), pBMB1653 (containing a 5.9 kb *Kpn*I-*Sac*I fragment of pBMB1652), pBMB1654 (containing a 4.8 kb *Sph*I-*Sac*I fragment of pBMB1652), pBMB165-F4A (deleting the fragment from position 3 to 532 of *orf4*), pBMB165-F5C (deleting the fragment from position 107 to the stop codon of *orf5*) and pBMB165-F6A (deletion of *orf6*), were able to replicate in *B. thuringiensis*. In contrast, the three other derivative plasmids pBMB1657 (containing a 3.0 kb *Hap*II fragment of pBMB1652), pBMB1658 (containing a 3.5 kb *Sca*I-*Sac*I fragment of pBMB1652) and pBMB1659 (containing a 3.9 kb *Sph*I-*Bgl*II fragment of pBMB1652) were unable to give rise to recombinant *B. thuringiensis *(Fig. 2).

Consequently, the pBMB165 mini-replicon (as harbored in pBMB165-F4A) was reduced to a 3.6 kb fragment with a single intact ORF, *orf1 *(*rep165*) encoding a 518-amino acids (aa) Rep protein (Rep165, in Fig. 2). Furthermore, a 266 bp fragment (from the *Sca*I site to the stop codon in *rep165*) was deleted by PCR amplification with the primer pair orf5-1 and orf5-4, which led the Rep165 protein to be truncated (deletion of 88 codons in *rep165*); and the corresponding construct pBMB165-F1B was unable to replicate in *B. thuringiensis *(Fig. 2).

Amino acid sequence comparison showed that Rep165 displayed similarity to the Rep proteins of the pAMβ1 family of theta-replicating plasmids, such as Rep of pBT9727 (97% identity), Rep63A of pAW63 (82% identity) and Rep of p43 (27% identity) from *B. thuringiensis *[8,14,15], RepS of pXO2 (81% identity) from *B. anthracis *[16], RepE of pAMβ1 (38% identity) from *Enterococcus faecalis *[10], RepR of pIP501 (38% identity) from *Streptococcus agalactiae *[12] and RepS of pSM19035 (38% identity) from *Streptococcus pyogenes *[13].

**Figure 3:**
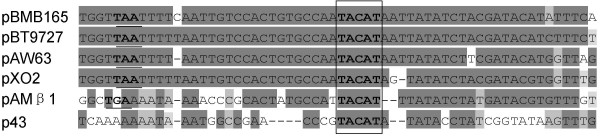
**Alignment of the origin of replication in pBMB165, pBT9727, pAW63, pXO2, pAMβ1 and p43**. The shaded letters indicate identical nucleotides. The box highlights a conserved replication initiation site 'TACAT'. The stop codons of the *rep *genes are underlined.

In addition, the minireplicon of pBMB165 was found to carry two *cis*-functioning regions. One *cis*-functioning region, located immediately downstream of *rep*165, displayed significant similarity to the *cis*-functioning origin of replication(*ori*) harbored in the corresponding locus of the pAMβ1 family plasmids cited above (Fig. 3). Moreover, a derivative of pBMB165-F5C (designated pBMB165-ORIB), in which only a 10 bp stretch remains of the original sequence located immediately downstream of *rep*165 (deleting most of the *ori165 *sequence), lost the ability to replicate in *B. thuringiensis *(Fig. 2). Based on the above observations, it can be concluded that this region carries the *ori *sequence (*ori165*) of pBMB165.

Taken together, the conservation among these Rep proteins and *ori *regions thus provided significant evidence that pBMB165 belongs to the pAMβ1 family of Gram-positive theta-replicating plasmids.

**Figure 4:**
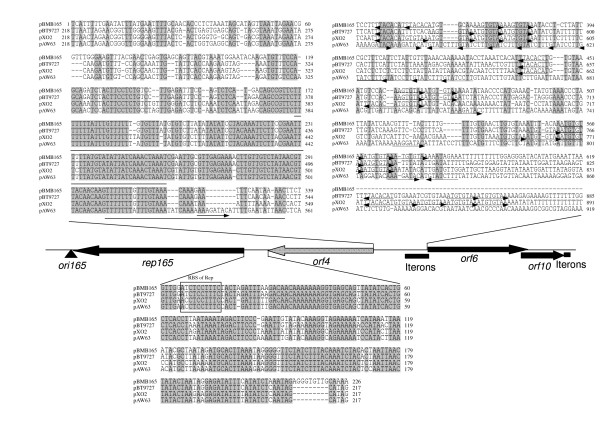
**Alignment of the non-coding regions between *rep *and replication-associated genes of pBMB165, pBT9727, pXO2 and pAW63**. The shaded letters indicate identical nucleotides. Arrows on the nucleotide sequences represent the iterons sequences. The conserved regions of high A+T content are underlined. The putative ribosome binding site (RBS) of *rep *is also highlighted in boldface.

**Figure 5:**
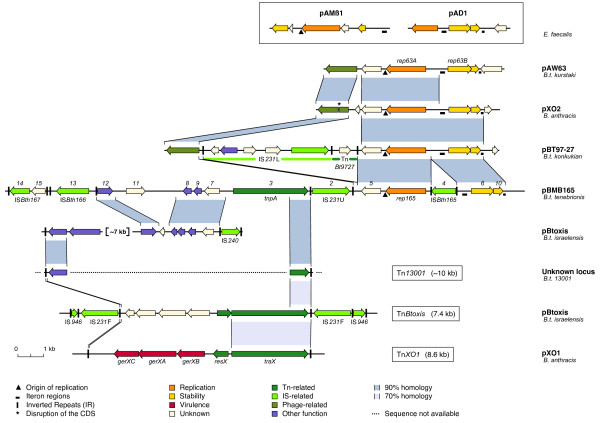
**Comparison of the 20 kb fragment carrying the pBMB165 replicon with relational fragments of other plasmids**. Predicted functions/homologies are shown by the color key featured below. The origins of replication, iteron regions and IRs are indicated.

The other *cis*-functioning region was constituted of iterons. These are sets of repeated DNA sequences that have been reported to serve as a binding site for the replication initiation protein and thus to play an important role in the replication and/or the control of replication in other theta-replicating plasmids [17]. In pBMB165, a set of 8 bp with the consensus sequence AT [A] GTGTAA was found repeated 7 times in the direct orientation and 3 times in reverse orientation, upstream of *rep165 *and immediately adjacent to *orf6*. Another three repeats were located downstream of *orf10*. The repeat sets were well conserved among pBMB165, pBT9727 and pXO2, although their reiterations were different, whereas they lacked any sequence homology to iterons from pAW63 (Fig. 4). It is noteworthy though that the iteron region was located in the equivalent loci on pBMB165, pXO2 and pBT927, as well as on the pAW63 replicon (Fig. 5). In addition, there were two conserved AT-rich DNA regions in the vicinity of the iterons sequences of pBMB165, pBT9727, pXO2 and pAW63 (Fig. 4).

To assess the involvement of these iterons in the replication of pBMB165, a deletion experiment was performed. The results obtained with pBMB165-F6B, which lacked the iterons, suggested that these repeated sequences are required for replication (Fig. 2). In contrast, the iterons sequences present upstream of *rep *from pAMβ1 have been shown to be non-essential to pAMβ1 replication [10].

### *orf6 *and *orf10 *are required for plasmid stability

As shown in Fig. 2, the recombinant plasmid pBMB1651 and its derivatives pBMB1652, pBMB1653, pBMB1654, pBMB165-F4A, pBMB165-F5C and pBMB165-F6A were unable to be properly maintained (after 40 generations at 30°C) in *B. thuringiensis *BMB171. This result showed that the 12 kb *EcoR*I replication fragment harbored only the factors required for replication and lacked some elements necessary for retaining plasmid stability.

To investigate the missing elements, a random YBT-1765 plasmid library was constructed by inserting *Bam*HI-partially digested plasmid DNA from YBT-1765 into the vector pBeloBAC11. In this small library, a recombinant plasmid, designated pBMB165B8, contained a 20 kb insert consisting of two *Bam*HI fragments of 16 kb and 4 kb; and further sequence analysis revealed that the 12 kb *Eco*RI replication region was located on the 16 kb *Bam*HI fragment (Fig. 2).

At the 3' end of this 20 kb fragment cloned from pBMB165, there were two intact overlapping putative ORFs, named *orf6 *and *orf10 *(Fig. 2), respectively. The same structural organization was also found in the corresponding location (between the two iterons) of plasmids pBT9727 (ORF47 and ORF48), pXO2 (ORF40 [*repB*] and ORF41) and pAW63 (ORF48 [*rep63B*] and ORF49) (Fig. 5).

ORF6 from pBMB165 shared sequence similarity with ORF47 (94% identity) from pBT9727 and RepB (ORF40, 89% identity) from pXO2, as well as copy control protein RepB (35% identity) from the *E. faecalis *plasmid pAD1 [18]. But while it resembled Rep63B (ORF48) from pAW63 in terms of location and size, they did not share any significant sequence similarity. These proteins, including Rep63B, were all shown to have conserved ATPase motif of ParA protein involved in chromosome or plasmid partitioning.

Likewise, ORF10 from pBMB165 displayed similarity to ORF48 from pBT9727 (86% identity) and ORF41 from pXO2 (66% identity), and although their predicted protein products did not display any sequence similarity to other known proteins, their size and location matched those of RepC, a protein involved in the stability of pAD1 [18], as did those of ORF49 from pAW63.

In order to conclusively identify the region responsible for the plasmid stability, the recombinant plasmid pBMB165-F6D (derived from pBMB165-F5C, additionally containing *orf6 *and *orf10*), and its derivatives pBMB165-F6E (obtained by deleting the fragment from position 311 to the stop codon of *orf10*) and pBMB165-F6G (a frame-shift mutation in *orf6 *due to the deletion of 7 bp from position 737 to 743) were constructed. The pBMB165-F6D construct was found to be highly stable in *B. thuringiensis *(almost 100 %), whereas pBMB165-F6E and pBMB165-F6G were not (Fig. 2), suggesting that *orf6 *and *orf10 *were both essential for plasmid stability.

Interestingly, previous studies have demonstrated that *repB *of pXO2 is not required for replication [16]. While in contrast, *rep63B *of pAW63 has previously been found to be indispensable for replication [8]. In this study, *orf6 *and *orf10 *were not essential for replication, but they were important for the stable maintenance of pBMB165. This result was consistent with previous observations on *repB *and *repC *of the *E. faecalis *plasmid pAD1, which is a member of another family of theta-replicating plasmids (group E). Nevertheless, the mechanism by which *orf6 *and *orf10 *contribute to plasmid stability control remains unclear.

### Transposable elements or their remnants

Three ORFs (*orf2*, *orf3 *and *orf4*) were found encoding putative transposases in the vicinity of *rep165 *(Fig. 5). An IS*231*-like element was identified downstream of *rep165 *and named IS*231*U. It harbored a single open reading frame encoding a 477-aa transposase (*orf2*) that displayed highest similarity to the transposases of IS*231*O-Q and F (81–87% identity).

Intriguingly, ORF3 (971-aa) and a downstream 59 bp sequence were similar to the transposase (TnpA) and right-side IR of several class II transposons from the *B. cereus *group, such as Tn*XO1 *(71 % TnpA identity) from *B. anthracis *pXO1, Tn*4430 *(39 % TnpA identity) and Tn*5401 *(26 % TnpA identity) from *B. thuringiensis *[19-21], as well as of an unnamed transposon from pBtoxis (ORFs pBt072 to pBt077, temporarily named Tn*Btoxis *in this paper) and a novel transposon (tentatively named Tn*13001*) from the *B. thuringiensis *subsp.*pakistani *strain T13001 (J. Mahillon, personal communication) (Fig. 5). These sequence features may be the transposase and right-side IR of a remnant class II transposon (tentatively named Tn*BMB165*). Furthermore, the IS*231*U described above had inserted just before the last bp (C) of this proposed IR, a peculiar structural association also seen in the case of IS*231*A insertion within Tn*4430 *[5], IS*231*F within Tn*Btoxis *and IS*231*L within Tn*Bt9727 *[22].

Another intact transposable element, designated IS*Bth165 *was found upstream of *rep165*. IS*Bth165 *contained a 289-aa putative transposase (ORF4) that displayed similarity with putative transposases from various bacterial species. It was tentatively grouped into the IS*5 *family of transposable elements [23]. It is particularly interesting to note that the replication control center of pBMB165 is divided into two parts by IS*Bth*165 (Fig. 4, Fig. 5): one part harboring *rep165 *and *ori165*, the other containing the iterons region and the two proposed stability-control elements (*orf6 *and *orf10*). Surprisingly, the presence of IS*Bth*165 in the intervening region between *rep165 *and *orf6 *apparently has little or no disruptive effect on the function of these replication-related factors (Fig. 4). An experiment involving the interruption of *orf4 *(pBMB165-F4A) showed that it was not implicated in plasmid replication (Fig. 2).

Finally, upstream of the replication region were two additional insertion sequences, designated IS*Bth*166 (with *orf13*) and IS*Bth*167 (with *orf14 *and *orf15*), that were tentatively classified as belonging to the IS*110 *and IS*3 *family of transposable elements [23], respectively, on the basis of their sequence similarities.

### Other ORFs

In addition to the replication/stability-related and transposable features, other elements were found in this cloned fragment. Downstream of the *rep165 *gene, an open reading frame (*orf5*) encoding a 250-aa hypothetical protein (Fig. 2) of unknown function displayed similarities with ORFs found in pBT9727, pXO2 and pAW63 in equivalent locations (Fig. 5).

The segment of pBMB165 spanning from *orf7 *to *orf12*, flanked on either side by transposable elements, was found to be remarkably similar to a corresponding segment on pBtoxis (ORFs pBt145 to pBt150). None of the sequences involved were found in pBT9727, pXO2 and pAW63 (Fig. 5). Based on their similarities, several of the genes carried by these segments were related to sporulation genes [24]. ORF12 of pBMB165 and pBt145 of pBtoxis were homologous to a putative spore coat-associated protein from *Bacillus*. ORF8 of pBMB165 and pBt148 of pBtoxis were homologous to the regulatory protein AbrB, which has been shown in *B. subtilis *to regulate sporulation [25], as well as being involved in an important example of chromosome-plasmid crosstalk in the *B. cereus *group [15,26]. ORF9 of pBMB165 and pBt149 of pBtoxis were homologous to members of the transcriptional regulator ArsR family.

### Sequence variability in the pAMβ1 family replicons

The pAMβ1 family replicons that have so far been identified from the *B. cereus *group (pBMB165, pBT9727, pXO2 and pAW63) have been shown to share the same organization (Fig. 5); however, this organization scheme is distinctly different from the one found in the pAMβ1 family replicons that are of enterococcal (pAMβ1) or streptococcal (pIP501 and pSM19035) origin (Fig. 5). The genetic divergence that led to the establishment of these two types of replicon apparently affected all the components upstream of the *rep *gene, including the iteron region and the stability-control genes of plasmids. It is of significant interest that the same organization of stability-associated genes found upstream of the *rep *genes in the *B. cereus *group replicons is also present in the group F family of theta-replicating plasmids (a novel family of theta plasmids from Gram-positive bacteria) which includes, most notably, plasmids pAD1 and pCF10 [27] from *E. faecalis*. It would therefore seem reasonable to speculate that a genetic exchange between the theta replicons of the group D family (pAMβ1 family) and those of the group F family may have been responsible for the evolution of the peculiar pAMβ1 family replicons found in the *B. cereus *group. For these reasons, we proposed that the pAMβ1 family replicons should be divided into two subfamilies with those from the *B. cereus *group forming a subfamily of their own.

The detailed sequence analysis performed on the pAMβ1 family replicons of the *B. cereus *group also showed that the iterons region and the two overlapping plasmid stability-associated genes constituted the highest source of sequence variability among these plasmid replicons, and that the major divergence from the general consensus was seen in the replicon of pAW63, a result consistent with the recent report of Van der Auwera *et al*. [22] which also suggested that these iterons region may act as recombination nodes participating in genetic plasticity.

## Conclusion

The mini-replicon of pBMB165 from *B. thuringiensis *subsp. *tenebrionis *YBT-1765 was determined. This study demonstrated that the determinants *rep165*, *ori165 *and the iterons region are indispensable for plasmid replication; and that *orf6 *and *orf10 *are involved in stable maintenance of the plasmid.

The exploration of sequence conservation among the pAMβ1 family replicons led to the suggestion that the pAMβ1 family replicons from the *B. cereus *group should be grouped into a subfamily that may have diverged mainly in the iterons and stability control regions. Comparative sequence analysis of additional such replicons may yield further insight into the genetic relationship of these divergent groups.

## Methods

### Bacteria, plasmids and media

*B. thuringiensis *subsp. *tenebrionis *YBT-1765 was isolated from a warehouse in China by our research group. The strain BMB171 was a plasmid-cured derivative of *B. thuringiensis *subsp. *kurstaki *YBT-1463 [28] isolated and preserved in our lab, which was used as the recipient strain in electroporation. The *Escherichia coli *TG1 strain (*supE*, *hsd*Δ5, *thi*, Δ (*lac-proAB*)/F' [*traD*36, *proAB*^+^, *lacI*^q^, *lacD*ΔM15]) was used for plasmid amplification. All strains were grown at 30 or 37°C in Luria-Bertani (LB) medium. Antibiotics were added at the following concentrations: ampicillin (100 μg/mL), kanamycin (50 μg/mL). Plasmid pUC19 was used for cloning and sequencing. Plasmid pDG780 was used as *B. thuringiensis *plasmid replicon cloning vector; it bears a replicon and an ampicillin resistance marker for selection in *E. coli *and a kanamycin resistance marker for selection in *Bacillus *[29].

### DNA manipulation

Plasmid DNA was prepared from *E. coli *strains by alkaline lysis procedure [30]. Plasmid DNA was obtained from *B. thuringiensis *strains by the modified method of Andrup *et al*. [31]. Plasmid DNA bands were separated by electrophoresis on a 0.8% agarose gel. Gel extraction of DNA was performed using AxyPrep DNA Gel Extraction Kit (Axygen Scientific, Inc).

DNA samples were electrophoresed in agarose gel and transferred to Hybond N+ (Amersham). Probe labeling was performed by the random primer method with digoxigenin according to the kit manufacture's protocol (Roche). Hybridization was carried out in 5 × SSC overnight at 65°C.

### Construction of recombinant plasmids

To determine the pBMB165 minireplicon, a number of deletion constructs were made by sub-cloning all deletion fragments into the vector pDG780 (Fig. 2). Because pDG780 lacked available restriction enzyme sites to clone some of these fragments, the vectors pUC19, pEG-28a (+) or pMD18-T Simple vector (a T-A cloning vector with no multiple cloning sites; TaKaRa Biotechnology [Dalian] Co., Ltd) were used to obtain sites suitable for cloning in pDG780. The details of the constructions are described below.

The vector pDG780 carrying the cloned 12 kb *Eco*RI fragment was designated pBMB1651 (Fig. 2). The 12 kb *Eco*RI fragment was partially digested with *Sau*3AI and ligated into pDG780 linearized with *Bam*HI. The ligation mixture was electroporated into the *B. thuringiensis *BMB171 strain. One transformant was obtained, and a plasmid preparation of this transformant was introduced into *E. coli *competent cells for further analysis. The resulting plasmid, named pBMB1652, harbored an 8.0 kb insert from pBMB1651 (Fig. 2).

A 5.9 kb *Kpn*I-*Sac*I fragment (*Sac*I site is present in the vector) from pBMB1652 was first inserted into pUC19, and then was cut by *Bam*HI and *Eco*RI and cloned into the corresponding sites of pDG780, generating plasmid pBMB1653. In the same way, a 3.0 kb *Hap*II fragment from pBMB1652 was sub-cloned into pUC19 at the site of *Acc*I, and then the fragment was digested with *Bam*HI and *Pst*I to be inserted into pDG780, resulting in plasmid pBMB1657. The insertion orientation of the 3.0 kb *Hap*II fragment was established by digestion with *Sca*I.

A 4.8 kb *Sph*I-*Sac*I fragment was cut from the plasmid pBMB1652 and the resulting 3' overhang of the *Sph*I site was rendered blunt with T4 DNA polymerase. The fragment was ligated into pDG780 between the *Sma*I and *Sac*I sites to form the plasmid pBMB1654. Plasmid pBMB1659 was constructed by ligating the 3.9 kb *Eco*RI-*Bgl*II fragment of pBMB1654 and *Eco*RI-*Bam*HI fragment of pDG780. Plasmid pBMB1658 was constructed by sub-cloning a 3.5 kb *Sca*I-*Sac*I fragment from the plasmid pBMB1652 into the *Sma*I-*Sac*I sites of pDG780.

A 598 bp fragment was amplified from pBMB1651 by the primer pair orf4-1 and orf6-2. The fragment was cloned into pBM1657 between the *Bam*HI and *Sac*I sites to generate plasmid pBMB165-F4A. A 704 bp fragment, contained the *Sca*I site in *rep165*, was amplified from pBMB1651 with the primer pair orf5-1 and orf5-2 and cloned into the vector pEG-28a (+) at the *Bam*HI and *Sac*I sites to produce plasmid pBMB165-F5A. Then, the 5.9 kb *Sca*I-*Sac*I pBMB165-F5A fragment and the 3.5 kb *Sca*I-*Sac*I pBMB1652 fragment were ligated to generate plasmid pBMB165-F5B. Finally, pBMB165-F5C was constructed by sub-cloning the 4.0 kb *Bam*HI-*Sac*I fragment of pBMB165-F5B into pDG780. Plasmids pBMB165-ORIB and pBMB165-F1B were constructed in the same manner.

The four fragments upstream of orf4 were amplified with the primer pairs orf6-1 and orf6-2, orf6-1 and orf6-3, orf6-1 and orf6-4, orf6-1 and orf6-5, respectively. The four amplified fragments were digested with *Bgl*II-*Sac*I and ligated to the 7.6 kb *Bgl*II-*Sac*I fragment of pBMB165-F5C, resulting in plasmids pBMB165-F6A, pBMB165-F6B, pBMB165-F6D and pBMB165-F6E, respectively.

Using the *Eco*RI site in orf6, a frame-shift mutation was introduced in orf6 by PCR amplification with the primer pair orf6-6 and orf6-4. The amplified fragment, deleted of the 7 bp following the *Eco*RI site in orf6, was digested with *Eco*RI-*Sac*I and ligated to the 4.0 kb *Eco*RI-*Sac*I fragment of pBMB165-F6T which was constructed by cloning the 1,974 bp fragment amplified with the primer pair orf6-1 and orf6-4 into the pMD18-T Simple vector, generating plasmid pBMB165-F6F. Then, a 1,926 bp *Bgl*II-*Sac*I fragment, cut from pBMB165-F6F, was ligated into the 7.6 kb *Bgl*II-*Sac*I fragment of pBMB165-F5C to generate plasmid pBMB165- F6G.

All the primers used are listed in Table 1. The high fidelity Ex-taq (TaKaRa Biotechnology [Dalian] Co., Ltd) DNA polymerase was used for amplification. PCR products were identified by sequencing.

### Transformation of *E. coli *and *B. thuringiensis*

Transformation of *E. coli *was carried out using CaCl_2_-treated competent cells, as described by Sambrook and Russell (2001). Transformation of *B. thuringiensis *plasmid-cured strain BMB171 was performed by electroporation with the Bio-Rad gene pulser set as previously described [32].

### Stability test

The stability of the recombinant plasmids in *B. thuringiensis *was tested under nonselective conditions at 30°C according to the method of Sanchis *et al*. [33]. One hundred single colonies of each recombinant were transferred onto LB plates and LB plates with kanamycin (50 μg/mL). Plasmid stability was estimated as the number of resistant colonies after about 40 generations. Each experiment was repeated three times.

### DNA sequence analysis

The replicon fragments of pBMB165 were sub-cloned into the vector pUC19 and DNA sequencing was performed using a primer walking strategy. Sequence homology searches were accomplished with a series of BLAST programs [34] in GenBank and EMBL sequence databases. Putative ORFs were predicted using ORF-finder [35] and Clone Manager 5 software. Multiple sequences were aligned based on Clustal W service at the European Bioinformatics Institute website [36] using the default parameters. For the comparative analysis among the plasmids, the sequences of pBT9727 [GenBank: CP000047], pAW63 [GenBank: DQ025752], pXO2 [GenBank: AE017335], pAMβ1 [GenBank: AF007787], p43 [GenBank: M60513], pBtoxis [GenBank: AL731825] and pAD1 [GenBank: L01794] were used. The nucleotide sequence of the pBMB165 replicon has been submitted to GenBank databases under the accession number DQ242517 [GenBank: DQ242517].

## Authors' contributions

JYH was responsible for experimental design, protocols and sequences analysis, and drafted the manuscript. SXG constructed the YBT-1765 plasmid library. JM critically revised the manuscript and coordinated the analysis of the transposable elements. GVdA provided constructive comments on the manuscript and participated in the analysis of transposable elements and preparation of figures. LW performed the study on restriction map of pBMB165. DMH participated in the sequence alignment. ZNY participated in revision of the manuscript and coordinated the study. MS conceived of the study, and participated in the design. All authors read and approved the final manuscript.

## Figures and Tables

**Table 1:** Primers used in this study

**Primer**	**Nucleotide sequence (5'-3')**	**Relevant characteristics**
orf4-1	5'-CGCGGATCCCATTCATTTTTGAATATTTATG-3'	forward primer, to construct pBMB165-F4A
orf5-1	5'-CGCGGATCCCTTCATGGCCAAGTAACTTAA-3'	forward primer, to construct pBMB165-F5C, pBMB165-F1B
orf5-2	5'-GGCGAGCTCCATGAGTGGGAAGCAGATATT-3'	reverse primer, to construct pBMB165-F5C, pBMB165-ORIB
orf5-3	5'-CGCGGATCCCAATTGAAAATTAACCAGCTG-3'	forward primer, to construct pBMB165-ORIB
orf5-4	5'-TCCCCCGGGATTTTCAATTGTCCACTGTGC-3'	reverse primer, to construct pBMB165-F1B
orf6-1	5'-AGCAGTTACGTAAATGGAATACAAGATG-3'	forward primer, to construct pBMB165-F6A, pBMB165-F6B, pBMB165-F6D, pBMB165-F6E
orf6-2	5'-CGAGAGCTCTACACATTTACACATTTACAC-3'	reverse primer, to construct pBMB165-F6A, pBMB165-F4A
orf6-3	5'-CGAGAGCTCACTTGTTGTAACGTTATAGAC-3'	reverse primer, to construct pBMB165-F6B
orf6-4	5'-CGAGAGCTCATTTATCTCTATACTGTGTTC-3'	reverse primer, to construct pBMB165-F6D, pBMB165-F6G
orf6-5	5'-CGAGAGCTCATAATCTTAGTTGTTCAGGTG-3'	reverse primer, to construct pBMB165-F6E
orf6-6	5'-CTGGAATTCTCCACGTGTTGGCTTAAATAC-3'	forward primer, to construct pBMB165-F6G
